# Localized and historical hypermobile spectrum disorders share self-reported symptoms and comorbidities with hEDS and HSD

**DOI:** 10.3389/fmed.2025.1594796

**Published:** 2025-08-13

**Authors:** DeLisa Fairweather, Katelyn A. Bruno, Ashley A. Darakjian, Frances C. Wilson, Jessica J. Fliess, Emma F. Murphy, S. Christian Kocsis, Max W. Strandes, Gabriel J. Weigel, Alayna M. Puls, Cameron J. Hartmoyer, Charwan Hamilton, Emily R. Whelan, Jessica M. Gehin, Stacey M. Menton, Hanna Sledge, David O. Hodge, Shilpa Gajarawala, Bala Munipalli, Chrisandra L. Shufelt, Paldeep S. Atwal, Dacre R. T. Knight

**Affiliations:** ^1^Department of Cardiovascular Medicine, Mayo Clinic, Jacksonville, FL, United States; ^2^Center for Clinical and Translational Science, Mayo Clinic, Rochester, MN, United States; ^3^Department of General Internal Medicine, Mayo Clinic, Jacksonville, FL, United States; ^4^Department of Immunology, Mayo Clinic, Jacksonville, FL, United States; ^5^Division of Cardiovascular Medicine, University of Florida, Gainesville, FL, United States; ^6^Department of Otolaryngology, Mayo Clinic, Jacksonville, FL, United States; ^7^Department of Quantitative Health Sciences, Mayo Clinic, Jacksonville, FL, United States; ^8^Atwal Clinic, West Palm Beach, FL, United States

**Keywords:** hypermobile Ehlers-Danlos syndrome, hypermobility spectrum disorders, localized hypermobility spectrum disorders, historical hypermobility spectrum disorders, diagnostic criteria, comorbidities, autism spectrum disorder

## Abstract

**Background:**

In 2017 a revised clinical criterion for the diagnosis of hypermobile Ehlers-Danlos syndrome (hEDS) was proposed in order to better distinguish hEDS from other joint hypermobility disorders which are termed hypermobility spectrum disorders (HSD). The goal of this study was to determine whether patients with localized HSD (L-HSD) or historical HSD (H-HSD) differed in 100 symptoms/comorbidities from controls and/or patients diagnosed with hEDS or HSD.

**Methods:**

In this study, we examined 100 self-reported symptoms/comorbidities from 2,695 patients diagnosed with hEDS, HSD, L-HSD/H-HSD, or controls.

**Results:**

From November 1, 2019, to August 27, 2024, 2,695 patients filled out an Intake Questionnaire at the Mayo Clinic Florida EDS Clinic. Using the 2017 diagnostic criterion, 60.6% (*n* = 1,632) of patients were diagnosed with HSD, 18.3% (*n* = 493) hEDS, 10.7% (*n* = 289) with L-HSD or H-HSD, and 10.4% (*n* = 281) were controls without any of these diagnoses. We found that patients with L-HSD/H-HSD self-reported significantly more symptoms/comorbidities than controls for 62/100 (62%) of issues compared to 58/100 (58%) for HSD and 20/100 (20%) for hEDS. These findings suggest that L-HSD/H-HSD share similar symptoms and comorbidities to HSD. Interestingly, patients with L-HSD/H-HSD self-reported significantly more symptoms/comorbidities than patients diagnosed with hEDS or HSD for 20/100 (20%) of issues such as joint pain, muscle weakness, multiple sensitivities, wheezing/shortness of breath, gastroesophageal reflux disease (GERD), pain/cramps in the lower abdomen, constipation, heat and/or cold intolerance, hearing difficulties, attention-deficit/hyperactivity disorder (ADHD), autism spectrum disorder (ASD), snoring, and narcolepsy. Symptoms/comorbidities that were significantly increased in L-HSD/H-HSD patients compared to controls (but not in hEDS or HSD compared to controls) and so were specific to this diagnosis included wheezing, hearing difficulties, narcolepsy, circadian rhythm disorders, and ASD.

**Conclusion:**

We found that patients with L-HSD/H-HSD had many symptoms and comorbidities that closely resembled HSD suggesting that revised diagnostic criteria for hEDS and HSD should include L-HSD/H-HSD within a diagnosis of HSD. Additionally, our data further suggest that patients with HSD (including L-HSD/H-HSD) have more symptoms/comorbidities than patients with hEDS.

## 1 Introduction

Hypermobile Ehlers-Danlos syndrome (hEDS) and hypermobility spectrum disorders (HSD) are heritable collagen disorders with widespread distribution of fragile connective tissue in the skin, joints, ligaments, and internal organs (1). 255 million people worldwide (3%) are estimated to have hEDS or HSD (2). Unlike the more classical forms of EDS, gene variants unique to hEDS/HSD are unknown except for a recent preprint report of mutations in the kallikrein gene family in patients with hEDS (3). Diagnosis is based on strict, physical criteria developed by the International EDS Consortium (Table 1) (4). Patients diagnosed with hEDS or HSD have been reported to have significantly greater joint pain, musculoskeletal pain, and fatigue than the general population (5). Comorbidities/symptoms of hypermobile patients are often multiorgan, multifaceted, and potentially debilitating (1, 2, 6).

**TABLE 1:** Diagnostic criteria for hypermobile EDS (hEDS) based on Malfait et al. (4).

Criteria 1 (GJH)	Criteria 2 (2 or more of A, B &/or C)	Criteria 3 (1–3 must be met)
GJH using the Beighton Score is ≥ 5 points out of 9 (wrists, pinkies, elbows, knees and hips)	A. Systemic manifestations of a more generalized connective tissue disorder (5/12 must be present)^a^	1. Absence of unusual skin fragility, which should prompt consideration of other types of EDS
Older patients: GJH using the Beighton Score is ≥ 4 points out of 9	B. Positive family history, with one or more first degree relatives independently meeting the current diagnostic criteria for hEDS	2. Exclusion of other heritable and acquired connective tissue disorders such as autoimmune conditions.^c^
	C. Musculoskeletal complications (must have at least one)^b^	3. Exclusion of alternative diagnoses that may include joint hypermobility by means of hypotonia and/or connective tissue laxity^d^

For a diagnosis of hEDS, criteria 1, 2 and 3 must be verified. ^a^1. Unusually soft or velvety skin; 2. Mild skin hyperextensibility; 3. Unexplained striae such as striae distensae or rubrae at the back, groins, thighs, breasts and/or abdomen in adolescents, men or prepubertal women without a history of significant gain or loss of body fat or weight; 4. Bilateral piezogenic papules of the heel; 5. Recurrent or multiple abdominal hernia(s) (e.g., umbilical, inguinal, crural); 6. Atrophic scarring involving at least two sites and without the formation of truly papyraceous and/or hemosideric scars as seen in classical EDS; 7. Pelvic floor, rectal, and/or uterine prolapse in children, men or nulliparous women without a history of morbid obesity or other known predisposing medical condition; 8. Dental crowding and high or narrow palate; 9. Arachnodactyly, as defined in one or more of the following: (i) positive wrist sign (Steinberg sign) on both sides; (ii) positive thumb sign (Walker sign) on both sides; 10. Arm span-to-height 1.05; 11. Mitral valve prolapse (MVP) mild or greater based on strict echocardiographic criteria; 12. Aortic root dilatation with Z-score > + 2. ^b^1. Musculoskeletal pain in two or more limbs, recurring daily for at least 3 months; 2. Chronic, widespread pain for > 3 months; 3. Recurrent joint dislocations or frank joint instability, in the absence of trauma (a or b): a. Three or more atraumatic dislocations in the same joint or two or more atraumatic dislocations in two different joints occurring at different times; b. Medical confirmation of joint instability at two or more sites not related to trauma. ^c^In patients with an acquired rheumatic connective tissue disorder (e.g., lupus, rheumatoid arthritis, etc.), additional diagnosis of hEDS requires meeting both Features A and B of Criterion 2. Feature C of Criterion 2 (chronic pain and/or instability) cannot be counted towards a diagnosis of hEDS in this situation. ^d^Alternative diagnoses and diagnostic categories include, but are not limited to, neuromuscular disorders (e.g., myopathic EDS, Bethlem myopathy), other HCTD (e.g., other types of EDS, Loeys–Dietz syndrome, Marfan syndrome), and skeletal dysplasias (e.g., OI). Exclusion of these considerations may be based upon history, physical examination, and/or molecular genetic testing, as indicated.

In 2017 a new clinical criterion for the diagnosis of hEDS was published that distinguished hEDS from HSD with a goal of better understanding the similarities and/or differences that exist within the spectrum of disease (4). The criteria for a diagnosis of hEDS briefly includes identification of generalized joint hypermobility (GJH) of specific joints using the Beighton Scale, evidence of a systemic connective tissue disorder, family history and/or musculoskeletal complications, and several exclusions (see Table 1) (4). The Beighton Scale assesses the joint hypermobility of only a few joints such as knees and elbows (4, 7). A Beighton Scale score of ≥ 5/9 after puberty or ≥ 4/9 after age 50 is a positive score (4). Patients are diagnosed with HSD if they do not meet the diagnostic criteria for hEDS, have a positive Beighton Scale score and also have evidence that the joint hypermobility is causing problems and it is not just an asymptomatic feature (feature C of the 2nd EDS criterion) (4, 7). Localized HSD (L-HSD) is diagnosed in patients that do not meet the diagnostic criteria for hEDS, do not have a positive Beighton Score (but their Beighton Score is not zero), and other areas of the body are hypermobile (7). Patients that do not meet the diagnostic criteria for hEDS, have a Beighton Score of zero, and experience hypermobility in other joints are termed historical HSD (H-HSD) (7).

In collaboration with the International Consortium’s working groups and the European Reference Networks, The Ehlers-Danlos Society aims to reassess the 2017 hEDS diagnostic criterion and formally define HSD (8). According to the Ehlers-Danlos Society website, part of their research goals include studying whether symptoms and comorbidities are similar and/or different between hEDS and HSD to determine whether the 2017 diagnostic criteria for hEDS should be revised (9, 10). Since the 2017 diagnostic criterion was established, several studies found either that symptoms and comorbidities between hEDS and HSD were essentially the same (9, 11–14) or that that both similarities and important differences exist (Table 2) (6, 8, 15, 16). A study by Aubry-Rozier et al. (13) examined 61 patients with hEDS and 36 with HSD using the 16-item Clinical Severity Score (CSS-16). They also assessed bone involvement, neuropathic pain (DN4) and symptoms of mast cell disorders (MCAS) as extra-articular manifestations. They found the two diagnoses were similar for most symptoms/comorbidities (Table 2) (13). They concluded from this study that patients with hEDS and HSD should receive the same clinical care. Martinez et al. examined 98 patients with hEDS and 27 with HSD using 7 health questionnaires (Short Form Health Survey (SF-36), Patient Health Questionnaire (PHQ)-15, Tampa Scale for Kinesiophobia (TSK), Fatigue Severity Scale (FSS), Epworth Sleepiness Scale (ESS), Gastro-Questionnaire, Composite Autonomic Symptom Score (COMPASS) 31) (14). They found that although a few differences between hEDS and HSD were observed, that the frequency and severity of most symptoms were indistinguishable between the two diagnoses (Table 2) (14). Ritelli et al. (8) examined 87 patients with hEDS and 126 with HSD for 90 symptoms/comorbidities and found that most occurred more often in patients with hEDS than HSD suggesting that hEDS may be more severe than HSD (Table 2) (8). Ritelli et al. (15) later reported a similar finding examining 9 symptoms/comorbidities in 94 patients with hEDS and 80 with HSD with more hEDS patients identified with these issues (Table 2) (15). Examining 423 patients with hEDS and 1,389 with HSD, we previously reported that most of 115 self-reported symptoms/comorbidities had extensive overlap between the two diagnoses, but that patients diagnosed with HSD reported more symptoms/comorbidities than patients with hEDS, suggesting that symptoms may be worse in patients with HSD (16). It is not clear why differences exist in the findings of different studies. Possible reasons include that most of the studies compared small numbers of patients (under 100/group), from different countries (i.e., races/ethnicities) and different ages (Table 2) (11–15). Importantly, none of these studies (aside from ours) indicated whether the patients diagnosed with HSD were HSD, L-HSD and/or H-HSD.

**TABLE 2:** Published studies comparing hEDS to HSD based on the 2017 diagnostic criteria.

Year/author	Country	hEDS^a^	HSD^a^	Key findings
Copetti et al. 2019 (11)	Italy	58 (36.7)	47 (?)^b^	This study found that patients with hEDS and HSD could both be divided into those with more severe vs. less severe disease based on **59 symptoms/comorbidities**. Thus, hEDS was not necessarily more severe than HSD.
Aubry-Rozier et al. 2021 (13)	Switzerland	61 (40.0)	36 (39.0)	This study found that patients with hEDS and HSD had similar severity scores for most of **16 symptoms/comorbidities** except for pain, motricity problems and spontaneous bleeding, and a similar spectrum of extra-articular manifestations. Patients with both diagnoses improved with physical therapy. They conclude that patient care should be the same.
Martinez et al. 2021 (14)	US	98 (37.8)	27 (40.9)	This study examined **7 symptom questionnaires** (i.e., gastrointestinal, autonomic dysfunction, etc.) and found that hEDS and HSD were worse than controls. Overall, they did not find significant differences between hEDS and HSD; however, this comparison was not made for all data.
Ritelli et al. 2022 (20)	Italy	20 (?)	20 (?)	This study conducted RNA sequencing of skin fibroblasts from patients and found that extracellular matrix gene profiles were present in both diagnoses. Verification of gene profiles found no significant difference in genes between the diagnoses.
Ritelli et al. 2024 (8)	Italy	87 (?)	126 (?)	This study examined around **90 symptoms/comorbidities** and found that patients with hEDS more frequently reported symptoms than HSD indicating differences between the two diagnoses and suggesting that hEDS patients may be more severe.
Darakjian et al. 2024 (16)	US	423 (33.8)	1,389 (34.8)	Our previous study examined **115 self-reported symptoms/comorbidities**. Several symptoms/comorbidities occurred at a high prevalence in both diagnoses like joint pain, allergy and headache/migraine. 9/115 (8%) symptoms/comorbidities were self-reported significantly more often in hEDS but 42/115 (37%) in HSD suggesting differences between the diagnoses and that HSD patients may be more severe.
Ritelli et al. 2025 (15)	Italy/US	94 (41.7)	80 (41.7)	This study examined **9 symptoms/comorbidities** and found that most occurred significantly more often in hEDS than HSD suggesting differences between the two diagnoses. However, they did not observe differences in serum autoimmune or extracellular matrix biomarkers between the two diagnoses.

^a^Data are shown as number of patients (mean age). ^b^A question mark indicates that the age of patients with that diagnosis was not clearly stated in the manuscript.

In previous manuscripts we had included L-HSD and H-HSD as part of our controls (patients attended the EDS Clinic but were not diagnosed with hEDS or HSD) (6, 17, 18). However, we published recently that patients with L-HSD/H-HSD were significantly different than controls that did not have any type of HSD for most of 115 symptoms/comorbidities (16). These findings led us to conduct this study. The goal of this study is to determine whether patients with L-HSD or H-HSD differ in 100 symptoms/comorbidities from controls and/or patients diagnosed with hEDS or HSD. An important issue that needs to be considered in future revisions of the EDS and HSD diagnostic criteria is the question of whether patients with HSD, L-HSD and/or H-HSD should be incorporated more fully into the diagnostic criteria. We found that patients with L-HSD/H-HSD had many symptoms and comorbidities that closely resembled HSD suggesting that revised diagnostic criteria for hEDS and HSD should include L-HSD/H-HSD within a diagnosis of HSD. Additionally, our data indicate that patients with HSD (including L-HSD/H-HSD) have more symptoms/comorbidities than patients with hEDS.

## 2 Materials and methods

### 2.1 Ethics statement

The Institutional Review Board (IRB# 19-010260) of Mayo Clinic approved the retrospective analysis of demographic and clinical data from medical records for this study and waived informed consent for all patients. The research conformed to the principles outlined in the Declaration of Helsinki.

### 2.2 Controls

Previously, we reported that patients seen at the EDS Clinic who were not diagnosed with HSD or hEDS using the 2017 diagnostic criteria were significantly different for most symptoms and comorbidities compared to diagnosed patients, (6) suggesting that this group may serve as appropriate controls. However, this control group contains patients diagnosed with L-HSD or H-HSD (7, 16). We published recently that patients with L-HSD/H-HSD were significantly different than controls that did not have any type of HSD for most of 115 symptoms/comorbidities (16). The goal of this study is to determine whether patients with L-HSD/H-HSD differ in 100 self-reported symptoms/comorbidities from controls and/or patients diagnosed with hEDS or HSD seen at the EDS Clinic.

### 2.3 Patients

Adult patients (≥ 18 years of age) were seen at the Mayo Clinic Florida EDS Clinic from November 1, 2019, to August 27, 2024 (*n* = 2,695) by self-referral or referrals from inside or outside Mayo Clinic. Patients were diagnosed with hEDS according to the 2017 diagnostic criteria (Table 1), (4) as previously (6, 16–18). hEDS does not have a recognized causative genetic variant compared to the other types of EDS (4). Briefly, the diagnostic criteria for hEDS includes identification of generalized joint hypermobility (GJH) of specific joints using the Beighton Scale (past puberty ≥ 5/9 and over 50 years of age ≥ 4/9), evidence of a systemic connective tissue disorder, family history and/or musculoskeletal complications, and several exclusions (Table 1) (4). HSD is diagnosed in patients that do not meet the diagnostic criteria for hEDS but have a positive Beighton Score (≥ 5/9 and ≥ 4/9 for older patients) and evidence that the joint hypermobility is causing problems and it is not just an asymptomatic feature (feature C of the 2nd EDS criterion) (4, 7). L-HSD is diagnosed in patients that do not meet the diagnostic criteria for hEDS, do not have a positive Beighton Score (but their Beighton Score was not zero), and other areas of the body are hypermobile (7). H-HSD is diagnosed in patients that do not meet the diagnostic criteria for hEDS, have a Beighton Score of zero, and other areas of the body are hypermobile (positive for the 5-point GJH questionnaire) (7, 19). The International Classification of Diseases, 10th Revision (ICD-10) codes used at Mayo Clinic for these conditions included: GJH (M24.80) (other specific joint derangements, unspecified site), hEDS (Q79.62), and HSD (M35.7) (hypermobility syndrome). HSD, L-HSD and H-HSD do not have specific ICD-10 codes, and M35.7 may be used for these conditions even though they have different diagnostic criteria (7). We track the specific EDS and HSD diagnosis of all patients seen at the EDS Clinic and so we were able to identify which patients received a diagnosis of L-HSD or H-HSD. Because we had very few H-HSD patients, we combined L-HSD and H-HSD patients for the analysis. Controls (*n* = 281) were patients that attended the EDS Clinic but were not diagnosed with hEDS (*n* = 493), HSD (*n* = 1,632), L-HSD or H-HSD (*n* = 289), as previously (16).

### 2.4 EDS Clinic data collection

Patient data were collected from November 1, 2019, to August 27, 2024 (*n* = 2,695). Patients received a 300-question REDCap Intake Questionnaire as standard of care prior to their first appointment at the Mayo Clinic Florida EDS Clinic. Self-reported data on 100 symptoms/comorbidities were obtained from the Intake Questionnaire of adult patients > 18 years of age. The Intake Questionnaire categorized questions by organ or system such as muscles, joints, allergy, neurological symptoms or gastrointestinal symptoms. Thus, data were organized in this manuscript according to those organ or system categories.

### 2.5 Statistical analysis

Continuous variables were summarized with the sample median and range. Categorical variables were summarized with number and percentage of subjects. Kruskal-Wallis rank sum test was used to compare the difference of continuous variables among groups. A χ^2^ test examined the association between two categorical variables. All the tests were two-tailed and *p* < 0.05 were considered statistically significant. All statistical analyses were performed using SAS version 9.4 (SAS Institute, Cary, NC, United States). Graphs were created using GraphPad Prism (Version 10.3.1).

## 3 Results

### 3.1 Patient demographics

From the 2,695 patients who were assessed for hypermobility at the EDS Clinic using the 2017 diagnostic criteria (Table 1) and the Morlino and Castori definitions of HSD, (4, 7) we found that 60.6% (*n* = 1,632) were diagnosed with HSD, 18.3% (*n* = 493) with hEDS, 10.7% (*n* = 289) with L-HSD or H-HSD, and 10.4% (*n* = 281) were controls without any of these diagnoses.

We found that the average age of controls was significantly older (38.7) than patients diagnosed with hEDS or HSD of any type (34.0–35.1) (*p* < 0.001) (Table 3). Over 85% in each group self-reported as females with the highest percentage of females in patients diagnosed with HSD (*p* < 0.001) (Table 3). Most patients in each category self-reported as not Hispanic White; however, the highest percentage of White was in patients with HSD (89%) and the lowest in hEDS (83%) (*p* = 0.015) (Table 3). More patients with L-HSD/H-HSD self-reported as Native Hawaii/Pacific Islander than for other diagnoses (*p* = 0.004) (Table 3). The BMI of controls (25.2) and patients with hEDS (24.8) was lower than patients with HSD (26.1) or L-HSD/H-HSD (26.4) (*p* < 0.001) (Table 4). Alcohol consumption was self-reported as highest in patients diagnosed with hEDS compared to other groups (*p* = 0.04) (Table 4). Where differences between groups occurred, the demographics of patients with L-HSD/H-HSD most closely resembled patients with a diagnosis of HSD rather than hEDS or controls.

**TABLE 3:** Demographics of patients diagnosed at the EDS Clinic (*n* = 2,695).

Attribute	Control (*n* = 281) number,%	hEDS (*n* = 493) number,%	HSD (*n* = 1,632) number,%	L-HSD/H-HSD (*n* = 289) number,%	*P*-value^a^
*Age, median (SD)*	38.7 (14.9)	35.1 (12.1)	34.0 (12.0)	35.0 (12.3)	< 0.001
*Patient sex*					< 0.001
Female	238 (84.7%)	437 (88.6%)	1517 (93.0%)	249 (86.2%)	
Male	42 (14.9%)	49 (9.9%)	79 (4.8%)	28 (9.7%)	
Non-binary	1 (0.4%)	6 (1.2%)	30 (1.8%)	11 (3.8%)	
Other	0 (0.0%)	1 (0.2%)	6 (0.4%)	1 (0.3%)	
*Race*					
American Indian/Alaskan native	2 (0.7%)	8 (1.6%)	26 (1.6%)	4 (1.4%)	0.71
Asian	4 (1.4%)	2 (0.4%)	27 (1.7%)	8 (2.8%)	0.06
Black or African American	7 (2.5%)	16 (3.2%)	37 (2.3%)	10 (3.5%)	0.50
Native Hawaii/Pacific Islander	0 (0.0%)	1 (0.2%)	3 (0.2%)	4 (1.4%)	0.004
White	243 (86.5%)	412 (83.6%)	1452 (89.0%)	252 (87.2%)	0.02
Other	1 (0.4%)	13 (2.6%)	43 (2.6%)	8 (2.8%)	0.13
Unknown/not disclosed	6 (2.1%)	7 (1.4%)	13 (0.8%)	5 (1.7%)	0.15
*Ethnicity*					< 0.001
Hispanic	13 (5.0%)	26 (5.9%)	123 (8.0%)	20 (7.1%)	
Not Hispanic	229 (88.1%)	403 (92.2%)	1377 (89.6%)	255 (90.7%)	
Unknown/not disclosed	18 (6.9%)	8 (1.8%)	37 (2.4%)	6 (2.1%)	
*Highest level of education*					0.56
Some high school	9 (3.5%)	7 (1.7%)	67 (4.4%)	10 (3.6%)	
High school/GED	21 (8.1%)	33 (8.0%)	129 (8.4%)	23 (8.2%)	
Some college	65 (25.2%)	97 (23.4%)	340 (22.2%)	61 (21.7%)	
Trade school	6 (2.3%)	18 (4.3%)	73 (4.8%)	12 (4.3%)	
Associate’s degree	22 (8.5%)	50 (12.1%)	166 (10.8%)	23 (8.2%)	
Bachelor’s degree	74 (28.7%)	118 (28.5%)	452 (29.5%)	85 (30.2%)	
Master’s degree	40 (15.5%)	64 (15.5%)	213 (13.9%)	52 (18.5%)	
Professional/Doctorate	17 (6.6%)	24 (5.8%)	82 (5.4%)	14 (5.0%)	
Not disclosed	4 (1.6%)	3 (0.7%)	9 (0.6%)	1 (0.4%)	

^a^*P*-values result from Fisher’s test for categorical data and Kruskal-Wallis rank sum test for numeric data.

**TABLE 4:** Self-reported socioenvironmental exposures (*n* = 2,695).

Attribute	Control (*n* = 281) number,%	hEDS (*n* = 493) number,%	HSD (*n* = 1,632) number,%	L-HSD/H-HSD (*n* = 289) number,%	*P*-value^a^
*BMI*					< 0.001
Mean (SD)	25.2 (5.3)	24.8 (5.3)	26.1 (5.7)	26.4 (5.7)	
Median	16.5–40.0	16.6–39.7	16.6–40.0	17.1–39.6	
*Smoking/Vaping history*					0.11
Yes-Currently	16 (6.2%)	46 (10.6%)	148 (9.6%)	34 (12.1%)	
Yes-Past	34 (13.1%)	85 (19.5%)	248 (16.1%)	53 (18.9%)	
No	207 (79.9%)	301 (69.2%)	1,129 (73.5%)	191 (68.0%)	
Unknown	2 (0.8%)	3 (0.7%)	12 (0.8%)	3 (1.1%)	
*Number of cigarettes smoked/day*					0.29
1-5	6 (12.0%)	21 (16.3%)	76 (19.2%)	18 (20.7%)	
6-10	7 (14.0%)	16 (12.4%)	46 (11.6%)	11 (12.6%)	
10-20	11 (22.0%)	11 (8.5%)	46 (11.6%)	15 (17.2%)	
> 20	3 (6.0%)	8 (6.2%)	13 (3.3%)	3 (3.4%)	
Unknown	12 (24.0%)	23 (17.8%)	65 (16.4%)	14 (16.1%)	
Vaping	11 (22.0%)	50 (38.8%)	150 (37.9%)	26 (29.9%)	
*Alcohol consumption*					0.04
Yes-currently	134 (51.7%)	238 (54.7%)	783 (50.9%)	124 (44.1%)	
Yes-past	62 (23.9%)	123 (28.3%)	403 (26.2%)	84 (29.9%)	
No	62 (23.9%)	70 (16.1%)	345 (22.4%)	72 (25.6%)	
Unknown	1 (0.4%)	4 (0.9%)	6 (0.4%)	1 (0.4%)	
*Number of alcoholic drinks consumed on average/week*					0.007
0-1	78 (39.8%)	135 (37.6%)	489 (41.2%)	79 (38.0%)	
2-3	36 (18.4%)	78 (21.7%)	191 (16.1%)	29 (13.9%)	
4-7	18 (9.2%)	34 (9.5%)	94 (7.9%)	21 (10.1%)	
7 +	7 (3.6%)	9 (2.5%)	34 (2.9%)	12 (5.8%)	
1-2 drinks/month	46 (23.5%)	85 (23.7%)	350 (29.5%)	55 (26.4%)	
Unknown	11 (5.6%)	18 (5.0%)	28 (2.4%)	12 (5.8%)	
*Alcohol exposure before birth*					0.96
Yes	6 (2.4%)	10 (2.4%)	28 (1.9%)	6 (2.2%)	
No	223 (87.8%)	359 (87.1%)	1313 (87.1%)	242 (88.3%)	
Unknown	25 (9.8%)	43 (10.4%)	166 (11.0%)	26 (9.5%)	
*Illicit drug use*					0.34
Yes-Currently	3 (1.2%)	15 (3.4%)	53 (3.5%)	10 (3.6%)	
Yes-Past	17 (6.6%)	40 (9.2%)	100 (6.5%)	21 (7.5%)	
No	231 (89.2%)	359 (82.3%)	1306 (85.0%)	236 (84.0%)	
Unknown/choose not to disclose	8 (3.1%)	22 (5.0%)	77 (5.0%)	14 (5.0%)	
*Exposure to drugs as a baby*					0.83
Yes	7 (2.7%)	10 (2.3%)	45 (3.0%)	8 (2.9%)	
No	221 (86.7%)	367 (85.7%)	1,299 (85.9%)	243 (88.7%)	
Unknown	27 (10.6%)	51 (11.9%)	169 (11.2%)	23 (8.4%)	

^a^P-values result from Chi-square test for categorical data and Kruskal-Wallis rank sum test for numeric data.

### 3.2 Joint and muscle weakness and easy bruising

For joint and muscle symptoms/comorbidities we found that patients with L-HSD/H-HSD self-reported more often than controls joint pain (*p* < 0.001), subluxations (*p* < 0.001), sprains (*p* < 0.001), easy bruising (*p* < 0.001), muscle weakness (*p* < 0.001), temporomandibular joint (TMJ) symptoms (*p* < 0.001), and dislocations (*p* < 0.001) (Table 5). Additionally, more L-HSD/H-HSD patients self-reported issues with joint pain (hEDS *p* < 0.001, HSD *p* = 0.037) and muscle weakness (hEDS *p* < 0.001, HSD *p* < 0.001) than patients diagnosed with hEDS or HSD (Table 5) indicating that L-HSD/H-HSD patients have significant joint and muscle symptoms/comorbidities.

**TABLE 5:** Comparison of joint and muscle symptoms/comorbidities (*n* = 2,695).

Symptoms/comorbidities^a^	Control (*n* = 281) number,%	hEDS (*n* = 493) number,%	HSD (*n* = 1,632) number,%	L-HSD/H-HSD (*n* = 289) number,%	*P*-value^b^
Joint pain	210 (74.7%)	410 (83.2%)**	1453 (89.0%)***	269 (93.1%)***^,∧^	< 0.001
Subluxations	138 (49.1%)	357 (72.4%)***	1180 (72.3%)***	210 (72.7%)***	< 0.001
Sprains	126 (44.8%)	330 (66.9%)***	1062 (65.1%)***	192 (66.4%)***	< 0.001
Easy bruising	124 (44.1%)	219 (44.4%)	949 (58.1%)***	178 (61.6%)***	< 0.001
Muscle weakness	122 (43.4%)	197 (40.0%)	848 (52.0%)**	184 (63.7%)***^,∧∧∧^	< 0.001
TMJ symptoms^c^	85 (30.2%)	177 (35.9%)	718 (44.0%)***	137 (47.4%)***	< 0.001
Dislocations	47 (16.7%)	182 (36.9%)***	484 (29.7%)***	88 (30.4%)***	< 0.001
No joint issues	31 (11.0%)	6 (1.2%)	27 (1.7%)	5 (1.7%)***	< 0.001

^a^Order of conditions based on highest percentage in patients with HSD. ^b^*P*-values result from Kruskal-Wallis rank sum test for numeric data. ^c^TMJ; temporomandibular joint dysfunction. *Compares control to hEDS, HSD or L-HSD/H-HSD, ^∧^compares HSD to L-HSD/H-HSD; ^∧^, *p* < 0.05; **, *p* < 0.01; *** or ^∧∧∧^, *p* < 0.001 by *t* test.

### 3.3 Asthma, allergy, and related symptoms

When we examined asthma and allergy symptoms, we found that all of the symptoms we examined were self-reported more often in patients with L-HSD/H-HSD than controls for allergy/atopy (*p* = 0.04), palpitations (*p* < 0.001), multiple sensitivities (*p* < 0.001), chest discomfort (*p* < 0.001), shortness of breath (*p* = 0.001), sun sensitivity (*p* < 0.001), hives (*p* = 0.039), rash (*p* = 0.048), oral ulcers (*p* = 0.001), and wheezing (*p* = 0.013) (Table 6). Additionally, more L-HSD/H-HSD patients self-reported issues with multiple sensitivities (hEDS *p* < 0.001, HSD *p* < 0.001), shortness of breath (hEDS *p* < 0.001, HSD *p* < 0.02), sun sensitivity (hEDS *p* < 0.001, HSD *p* < 0.008), and wheezing (hEDS *p* = 0.001, HSD *p* < 0.01) than patients diagnosed with hEDS or HSD (Table 6) indicating that patients with L-HSD/H-HSD have significant asthma/allergy symptoms and comorbidities.

**TABLE 6:** Comparison of asthma, allergy, and potentially related symptoms/comorbidities (*n* = 2,695).

Symptoms/comorbidities^a^	Control (*n* = 281) number,%	hEDS (*n* = 493) number,%	HSD (*n* = 1,632) number,%	L-HSD/H-HSD (*n* = 289) number,%	*P*-value^b^
Allergies/atopy	90 (67.2%)	181 (77.0%)	433 (77.0%)*	26 (86.7%)*	0.04
Palpitations	89 (31.7%)	182 (36.9%)	734 (45.0%)***	128 (44.3%)**	< 0.001
Multiple sensitivities (lights, smells, foods, medicine)	99 (35.2%)	169 (34.3%)	718 (44.0%)**	174 (60.2%)***^,∧∧∧^	< 0.001
Chest discomfort	76 (27.0%)	151 (30.6%)	655 (40.1%)***	132 (45.7%)***	< 0.001
Shortness of breath	68 (24.2%)	139 (28.2%)	589 (36.1%)***	125 (43.3%)***^,∧^	< 0.001
Sun sensitivity	64 (22.8%)	106 (21.5%)	472 (28.9%)*	106 (36.7%)***^,∧∧^	< 0.001
Hives	41 (14.6%)	83 (16.8%)	335 (20.5%)*	62 (21.5%)*	0.04
Rash	45 (16.0%)	81 (16.4%)	327 (20.0%)	67 (23.2%)*	0.05
Oral ulcers	33 (11.7%)	60 (12.2%)	296 (18.1%)**	56 (19.4%)*	0.001
Wheezing	26 (9.3%)	38 (7.7%)	160 (9.8%)	43 (14.9%)*^,∧^	0.01

^a^Order of conditions based on highest percentage in patients with HSD. ^b^*P*-values obtained from Kruskal-Wallis rank sum test. *Compares control to hEDS, HSD or L-HSD/H-HSD, ^∧^compares HSD to L-HSD/H-HSD; * or ^∧^, *p* < 0.05; ** or ^∧∧^, *p* < 0.01; *** or ^∧∧∧^, *p* < 0.001 by *t* test.

### 3.4 Neurological symptoms and comorbidities

When we examined neurological symptoms and comorbidities, we found that all of the symptoms we examined were self-reported more often in patients with L-HSD/H-HSD than controls including brain fog (*p* < 0.001), headache (*p* < 0.001), light-headedness (*p* < 0.001), numbness/tingling of extremities (*p* < 0.001), migraine (*p* < 0.001), pain/cramps in lower abdomen (*p* < 0.001), sense of imbalance (*p* < 0.001), heat intolerance (*p* < 0.001), palpitations (*p* < 0.001), cold intolerance (*p* < 0.001), multiple sensitivities (*p* < 0.001), ringing in the ears (*p* = 0.003), vertigo (*p* = 0.001), blurred vision (*p* = 0.013), dry eyes (*p* = 0.005), increased sweating (*p* < 0.001), dry mouth (*p* < 0.001), autonomic dysfunction (*p* = 0.002), new daily persistent headache (*p* = 0.002), ADD/ADHD (*p* = 0.011), chronic migraine (*p* = 0.005), hearing difficulties (*p* = 0.002), and ASD (*p* < 0.001) (Table 7). Additionally, more L-HSD/H-HSD patients self-reported issues with pain/cramps in lower abdomen (hEDS *p* < 0.001, HSD *p* = 0.026), heat intolerance (hEDS *p* < 0.001, HSD *p* < 0.014), cold intolerance (hEDS *p* < 0.001, HSD *p* < 0.018), multiple sensitivities (hEDS *p* < 0.001, HSD *p* < 0.001), ADD/ADHD (hEDS *p* = 0.014, HSD *p* = 0.047), hearing difficulties (hEDS *p* < 0.001, HSD *p* = 0.026), and ASD (hEDS *p* = 0.005, HSD *p* = 0.002) than patients diagnosed with hEDS or HSD (Table 7) indicating that patients with L-HSD/H-HSD have significant neurological symptoms/comorbidities.

**TABLE 7:** Comparison of neurologic symptoms/comorbidities (*n* = 2,695).

Symptoms/comorbidities^a^	Control (*n* = 281) number,%	hEDS (*n* = 493) number,%	HSD (*n* = 1,632) number,%	L-HSD/H-HSD (*n* = 289) number,%	*P*-value^b^
Brain fog	172 (61.2%)	349 (70.8%)**	1215 (74.4%)***	225 (77.9%)***	< 0.001
Headache	149 (53.0%)	342 (69.4%)***	1124 (68.9%)***	205 (70.9%)***	< 0.001
Light-headedness	128 (45.6%)	234 (47.5%)	983 (60.2%)***	189 (65.4%)***	< 0.001
Numbness/tingling of extremities	122 (43.4%)	205 (41.6%)	961 (58.9%)***	182 (63.0%)***	< 0.001
Migraine	105 (37.4%)	266 (54.0%)***	852 (52.2%)***	165 (57.1%)***^,^	< 0.001
Pain/cramps in lower abdomen	106 (37.7%)	199 (40.4%)	856 (52.5%)***	172 (59.5%)***^,∧^	< 0.001
Sense of imbalance	105 (37.4%)	182 (36.9%)	817 (50.1%)***	157 (54.3%)***	< 0.001
Heat intolerance	91 (32.4%)	169 (34.3%)	748 (45.8%)***	155 (53.6%)***^,∧^	< 0.001
Palpitations	89 (31.7%)	182 (36.9%)	734 (45.0%)***	128 (44.3%)**	< 0.001
Cold intolerance	98 (34.9%)	194 (39.4%)	730 (44.7%)**	151 (52.2%)***^,∧^	< 0.001
Multiple sensitivities (lights, smells, foods, medicine)	99 (35.2%)	169 (34.3%)	718 (44.0%)**	174 (60.2%)***^,∧∧∧^	< 0.001
Ringing in the ears	100 (35.6%)	171 (34.7%)	692 (42.4%)*	129 (44.6%)*	0.003
Vertigo	72 (25.6%)	174 (35.3%)**	616 (37.7%)***	106 (36.7%)**	0.001
Blurred vision	89 (31.7%)	149 (30.2%)	604 (37.0%)	112 (38.8%)	0.01
Dry eyes	88 (31.3%)	148 (30.0%)	603 (36.9%)	116 (40.1%)*	0.005
Increased sweating	68 (24.2%)	126 (25.6%)	569 (34.9%)***	102 (35.3%)**	< 0.001
Dry mouth	68 (24.2%)	125 (25.4%)	509 (31.2%)*	114 (39.4%)***	< 0.001
Autonomic dysfunction	61 (21.7%)	172 (34.9%)***	479 (29.4%)**	87 (30.1%)*	0.002
New daily persistent headache	50 (17.8%)	121 (24.5%)*	464 (28.4%)***	77 (26.6%)*	0.002
ADD/ADHD^c^	63 (22.4%)	128 (26.0%)	465 (28.5%)*	99 (34.3%)**^,∧^	0.01
Chronic migraine	48 (17.1%)	126 (25.6%)**	433 (26.5%)***	83 (28.7%)***	0.005
Hearing difficulties	53 (18.9%)	84 (17.0%)	360 (22.1%)	81 (28.0%)*^,∧^	0.002
Autism spectrum disorder (ASD)	15 (5.3%)	36 (7.3%)	120 (7.4%)	40 (13.8%)***^,∧∧∧^	< 0.001

^a^Order of conditions based on highest percentage in patients with HSD. ^b^*P*-values obtained from Kruskal-Wallis rank sum test. ADD; attention deficit disorder; ADHD attention deficit hyperactivity disorder; ASD, autism spectrum disorder. *Compares control to hEDS, HSD or L-HSD/H-HSD, ^∧^compares HSD to L-HSD/H-HSD; * or ^∧^, *p* < 0.05; **, *p* < 0.01; *** or ^∧∧∧^, *p* < 0.001 by *t*-test.

### 3.5 Gastrointestinal symptoms

When we examined gastrointestinal symptoms and comorbidities, we found that over half of the symptoms we examined were self-reported more often in patients with L-HSD/H-HSD than controls including pain/cramps in the lower abdomen (*p* < 0.001), diarrhea (*p* < 0.001), constipation (*p* < 0.001), bowel cramps (*p* < 0.001), GERD (*p* = 0.005), irritable bowel syndrome (IBS) (*p* = 0.035), dyspepsia (*p* = 0.009), loss of appetite (*p* < 0.001), frequent loose stools (*p* < 0.001), vomiting (*p* = 0.001), hemorrhoids (*p* = 0.042), and rectal prolapse (*p* = 0.02) (Table 8). Additionally, more L-HSD/H-HSD patients self-reported issues with pain/cramps in lower abdomen (hEDS *p* < 0.001, HSD *p* = 0.026), constipation (hEDS *p* < 0.001, HSD *p* = 0.008), bowel cramps (hEDS *p* < 0.001, HSD *p* = 0.039), and GERD (hEDS *p* = 0.012, HSD *p* = 0.036) than patients diagnosed with hEDS or HSD (Table 8) indicating that patients with L-HSD/H-HSD have significant gastrointestinal symptoms/comorbidities.

**TABLE 8:** Comparison of gastrointestinal symptoms/comorbidities (*n* = 2,695).

Symptoms/comorbidities^a^	Control (*n* = 281) number,%	hEDS (*n* = 493) number,%	HSD (*n* = 1,632) number,%	L-HSD/H-HSD (*n* = 289) number,%	*P*-value^b^
Nausea	108 (38.4%)	221 (44.8%)***	909 (55.7%)***	168 (58.1%)***	< 0.001
Pain/cramps in lower abdomen	106 (37.7%)	199 (40.4%)	856 (52.5%)***	172 (59.5%)***^,∧^	< 0.001
Diarrhea	105 (37.4%)	245 (49.7%)***	846 (51.8%)***	161 (55.7%)***	< 0.001
Constipation	95 (33.8%)	177 (35.9%)*	793 (48.6%)***	165 (57.1%)***^,∧∧∧^	< 0.001
Bowel cramps	86 (30.6%)	190 (38.5%)*	768 (47.1%)***	155 (53.6%)***^,∧^	< 0.001
GERD (Reflux)^c^	95 (33.8%)	192 (38.9%)	677 (41.5%)*	139 (48.1%)***^,∧^	0.005
Irritable bowel syndrome (IBS)	77 (27.4%)	156 (31.6%)	580 (35.5%)**	102 (35.3%)*	0.04
Dyspepsia (heartburn)	75 (26.7%)	146 (29.6%)	571 (35.0%)**	104 (36.0%)*	0.009
Loss of appetite	68 (24.2%)	131 (26.6%)	570 (34.9%)***	111 (38.4%)***	< 0.001
Frequent loose stools	58 (20.6%)	145 (29.4%)**	543 (33.3%)***	107 (37.0%)***	< 0.001
Vomiting	54 (19.2%)	146 (29.6%)**	503 (30.8%)***	91 (31.5%)***	0.001
Hemorrhoids	67 (23.8%)	158 (32.0%)*	442 (27.1%)	89 (30.8%)	0.04
Gastroparesis	44 (15.7%)	97 (19.7%)	303 (18.6%)	49 (17.0%)	0.50
Fissure (tear in anus)	27 (9.6%)	62 (12.6%)	176 (10.8%)	35 (12.1%)	0.54
Fecal incontinence	16 (5.7%)	33 (6.7%)	76 (4.7%)	18 (6.2%)	0.28
Rectal prolapse	13 (4.6%)	40 (8.1%)	75 (4.6%)	18 (6.2%)	0.02
Barrett’s esophagus	7 (2.5%)	7 (1.4%)	34 (2.1%)	7 (2.4%)	0.69
Ulcerative colitis	6 (2.1%)	7 (1.4%)	28 (1.7%)	10 (3.5%)	0.19
Crohn’s disease	2 (0.7%)	7 (1.4%)	18 (1.1%)	4 (1.4%)	0.81

^a^Order of conditions based on highest percentage in patients with HSD. ^b^P-values obtained from Kruskal-Wallis rank sum test. ^c^GERD, gastroesophageal reflux disease; GI, gastrointestinal. *Compares control to hEDS, HSD or L-HSD/H-HSD, ^∧^compares HSD to L-HSD/H-HSD; * or ^∧^, *p* < 0.05; **, *p* < 0.01; *** or ^∧∧∧^, *p* < 0.001 by *t* test.

### 3.6 Sleep symptoms and comorbidities

When we examined sleep symptoms and comorbidities, we found that half of the symptoms we examined were self-reported more often in patients with L-HSD/H-HSD than controls including difficulty falling and staying asleep (*p* < 0.001), insomnia (*p* = 0.005), idiopathic hypersomnia (*p* = 0.032), snoring (*p* < 0.001), narcolepsy (*p* = 0.042), and circadian rhythm disorders (*p* < 0.001) (Table 9). Additionally, more L-HSD/H-HSD patients self-reported issues with snoring (hEDS *p* < 0.001, HSD *p* < 0.001) and narcolepsy (hEDS *p* = 0.14, HSD *p* = 0.006) than patients diagnosed with hEDS or HSD (Table 9) indicating that patients with L-HSD/H-HSD have some sleep symptoms and comorbidities.

**TABLE 9:** Comparison of sleep symptoms/comorbidities (*n* = 2,695).

Symptoms/comorbidities^a^	Control (*n* = 281) number,%	hEDS (*n* = 493) number,%	HSD (*n* = 1,632) number,%	L-HSD/H-HSD (*n* = 289) number,%	*P*-value^b^
Difficulty falling asleep and staying asleep	144 (51.2%)	226 (45.8%)	972 (59.6%)**	189 (65.4%)***	< 0.001
Insomnia	94 (33.5%)	202 (41.0%)*	687 (42.1%)**	139 (48.1%)***	0.005
Sleep disturbances	94 (33.5%)	196 (39.8%)	645 (39.5%)	105 (36.3%)	0.20
Restless leg syndrome	58 (20.6%)	118 (23.9%)	394 (24.1%)	76 (26.3%)	0.46
Idiopathic hypersomnia	37 (13.2%)	79 (16.0%)	321 (19.7%)*	55 (19.0%)*	0.03
Snoring	22 (7.8%)	50 (10.1%)	200 (12.3%)*	59 (20.4%)***^,∧∧∧^	< 0.001
Obstructive sleep apnea	24 (8.5%)	41 (8.3%)	138 (8.5%)	37 (12.8%)	0.11
Narcolepsy	11 (3.9%)	26 (5.3%)	69 (4.2%)	23 (8.0%)*^,∧∧^	0.04
Parasomnia	3 (1.1%)	14 (2.8%)	42 (2.6%)	11 (3.8%)	0.22
Circadian rhythm disorders	5 (1.8%)	9 (1.8%)	36 (2.2%)	19 (6.6%)**^,∧∧∧^	< 0.001

^a^Conditions listed by highest percentage in patients with HSD. ^b^*P*-values result from Kruskal-Wallis rank sum test for numeric data. *Compares control to hEDS, HSD or L-HSD/H-HSD, ^∧^compares HSD to L-HSD/H-HSD; *, *p* < 0.05; ** or ^∧∧^, *p* < 0.01; *** or ^∧∧∧^, *p* < 0.001 by *t* test.

### 3.7 Psychological conditions and abuse

When we examined psychological symptoms and comorbidities, we found that over half of the symptoms we examined were self-reported more often in patients with L-HSD/H-HSD than controls including anxiety (*p* < 0.001), depression (*p* < 0.001), depressed mood (*p* < 0.001), nervousness (*p* < 0.001), abuse (*p* = 0.042), emotional/verbal abuse (*p* < 0.001), PTSD (*p* = 0.001), ADD/ADHD (*p* = 0.011), sexual abuse (*p* = 0.038), and ASD (*p* < 0.001) (Table 10). As described above, more L-HSD/H-HSD patients self-reported issues with ADD/ADHD (hEDS *p* = 0.014, HSD *p* = 0.047) and ASD (hEDS *p* = 0.005, HSD *p* = 0.002) than patients diagnosed with hEDS or HSD (Table 10) indicating that patients with L-HSD/H-HSD may have more of these conditions. However, none of the other psychological symptoms/comorbidities or abuse that we examined were elevated in patients with L-HSD/H-HSD.

**TABLE 10:** Comparison of psychological symptoms/comorbidities (*n* = 2,695).

Symptoms/comorbidities^a^	Control (*n* = 281) number,%	hEDS (*n* = 493) number,%	HSD (*n* = 1,632) number,%	L-HSD/H-HSD (*n* = 289) number,%	*P*-value^b^
Anxiety	159 (56.6%)	306 (62.1%)	1137 (69.7%)***	192 (66.4%)*	< 0.001
Depression	121 (43.1%)	262 (53.1%)**	953 (58.4%)***	164 (56.7%)***	< 0.001
Depressed mood	95 (33.8%)	159 (32.3%)	744 (45.6%)***	137 (47.4%)***	< 0.001
Nervousness	97 (34.5%)	156 (31.6%)	727 (44.5%)**	133 (46.0%)**	< 0.001
Abuse	69 (27.0%)	154 (35.6%)	590 (39.0%)***	114 (41.6%)***	< 0.001
Emotional/verbal abuse	54 (19.2%)	123 (24.9%)	491 (30.1%)***	90 (31.1%)***	< 0.001
PTSD^c^	55 (19.6%)	119 (24.1%)	461 (28.2%)**	95 (32.9%)***	0.001
ADD/ADHD	63 (22.4%)	128 (26.0%)	465 (28.5%)*	99 (34.3%)**^,∧^	0.011
Sexual abuse	41 (14.6%)	93 (18.9%)	344 (21.1%)*	67 (23.2%)**	0.04
Physical abuse	40 (14.2%)	79 (16.0%)	300 (18.4%)	55 (19.0%)	0.25
Obsessive compulsive disorder	34 (12.1%)	84 (17.0%)	264 (16.2%)	49 (17.0%)	0.28
Eating disorders	25 (8.9%)	59 (12.0%)	223 (13.7%)	28 (9.7%)	0.06
Body dysmorphic disorder	16 (5.7%)	27 (5.5%)	135 (8.3%)	21 (7.3%)	0.13
Autism/autism spectrum disorder	15 (5.3%)	36 (7.3%)	120 (7.4%)	40 (13.8%)***^,∧∧∧^	< 0.001
Bipolar	12 (4.3%)	27 (5.5%)	103 (6.3%)	14 (4.8%)	0.46
Personality disorder	7 (2.5%)	8 (1.6%)	49 (3.0%)	9 (3.1%)	0.40
Conversion disorder	2 (0.7%)	7 (1.4%)	21 (1.3%)	1 (0.3%)	0.44
Schizophrenia	0 (0.0%)	0 (0.0%)	4 (0.2%)	0 (0.0%)	0.46

*^a^*Conditions are listed in order of highest percentage in patients diagnosed with HSD. ^b^ *P*-values result from Kruskal-Wallis rank sum test for numeric data. ^c^ADD, attention deficit disorder; ADHD, attention deficit hyperactivity disorder; PTSD, post-traumatic stress disorder. *Compares control to hEDS, HSD or L-HSD/H-HSD, ^∧^compares HSD to L-HSD/H-HSD; * or ^∧^, *p* < 0.05; **, *p* < 0.01; *** or ^∧∧∧^, *p* < 0.001 by *t*-test.

## 4 Discussion

**FIGURE 1:**
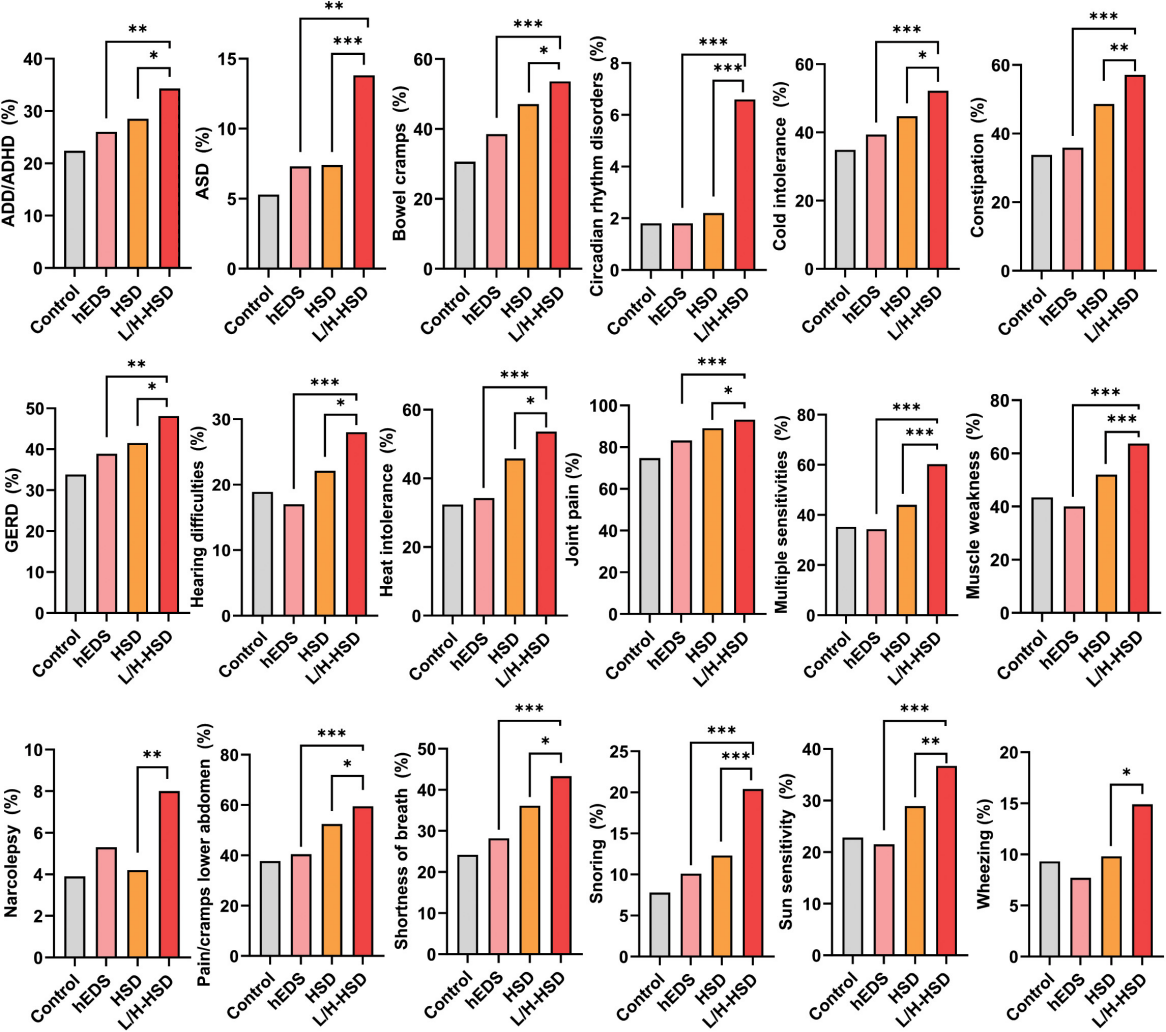
Symptoms and comorbidities that were significantly increased in patients with L-HSD/H-HSD compared to hEDS or HSD. Twenty out of 100 or 20% of the self-reported symptoms and comorbidities examined in this study were significantly higher in patients with L-HSD/H-HSD compared to other diagnoses indicating that these patients should be incorporated into the diagnostic criteria for HSD in the future (not all shown). *p < 0.05, **p < 0.01, ***p < 0.001 using Chi-square test comparing hEDS or HSD to L/H-HSD. Each graph was significant comparing 4 groups- see tables.

To our knowledge, this is the first study to compare symptoms/comorbidities of L-HSD/H-HSD to controls or to patients diagnosed with hEDS and HSD. In this study, we found that patients with L-HSD/H-HSD had symptoms/comorbidities that closely resembled HSD suggesting that revised diagnostic criteria for hEDS and HSD should include L-HSD/H-HSD in the HSD diagnosis. Most of the symptoms/comorbidities that we examined in this study occurred in all patient groups; however, patients with HSD and L-HSD/H-HSD self-reported more symptoms/comorbidities than those diagnosed with hEDS or controls suggesting that patients with HSD, L-HSD and H-HSD may have more severe disease than patients with hEDS. We found that patients with L-HSD/H-HSD self-reported significantly more symptoms/comorbidities than controls for 62/100 (62%) of issues compared to 58/100 (58%) for HSD and 20/100 (20%) for hEDS. These findings indicate that L-HSD/H-HSD share similar symptoms and comorbidities to HSD and differ from hEDS and controls. Importantly, patients with L-HSD/H-HSD self-reported significantly more symptoms/comorbidities than patients diagnosed with hEDS or HSD for 20/100 (20%) of issues. These symptoms/comorbidities included joint pain, muscle weakness, multiple sensitivities, wheezing/shortness of breath, GERD, pain/cramps in the lower abdomen, constipation, heat and/or cold intolerance, hearing difficulties, ADHD, ASD, snoring, and narcolepsy (Figure 1). Additionally, some symptoms/comorbidities were self-reported exclusively more often in L-HSD/H-HSD patients compared to controls, but not in hEDS or HSD compared to controls, including wheezing, hearing difficulties, narcolepsy, circadian rhythm disorders, and ASD (Figure 1). These findings suggest that patients with L-HSD/H-HSD may have more symptoms/comorbidities than hEDS and HSD patients using current diagnostic definitions. However, it is possible that individuals diagnosed with L-HSD or H-HSD may be less likely to present to a specialty clinic unless they experience significant systemic symptoms (e.g., dysautonomia, GI distress, or neurocognitive issues). This referral or selection bias could contribute to the unexpectedly high symptom burden in this group relative to patients with hEDS or HSD, who may be referred based on more classical joint findings alone. Interestingly, patients with hEDS more closely resembled controls for many symptoms/comorbidities. Controls attended the EDS Clinic because they believed they could have hEDS but did not receive the diagnosis of hEDS, HSD, L-HSD or H-HSD. The controls consistently had fewer symptoms/comorbidities than HSD and L-HSD/H-HSD patients, but we do not know what their appropriate diagnosis is.

Since the development of the 2017 diagnostic criterion, a number of studies have compared hEDS to HSD to determine whether the two diagnoses represent a spectrum or are two distinct conditions. A study by Copetti et al. examined 105 patients diagnosed with hEDS or HSD for 59 characteristics and analyzed data using hierarchical clustering of principal components (11). They found that some patients within both diagnoses were considered more severe regardless of diagnosis and recommended a new severity score. This finding is similar to our previous report that around 70% of the patients seen at the Mayo Clinic Florida EDS Clinic were diagnosed with fibromyalgia, a condition characterized by widespread joint and muscle pain (6). This 70% included patients with L-HSD/H-HSD. We found that patients diagnosed with hEDS or HSD that also had a diagnosis of fibromyalgia (56% of patients) self-reported significantly more symptoms and comorbidities than those without a fibromyalgia diagnosis suggesting more severe disease (6). Copetti et al. (11) did not indicate whether the patients with greater severity in their study also had a diagnosis of fibromyalgia (11). Perhaps future revised hEDS/HSD diagnosis criteria could include an assessment/diagnosis of fibromyalgia.

We recently examined 2,088 patients at the Mayo Clinic Florida EDS Clinic using the 2017 diagnostic criteria comparing 115 self-reported symptoms/comorbidities between hEDS, HSD and controls to determine similarities and differences between the diagnoses (16). We found that all patients experienced some level of these symptoms/comorbidities and they were reported significantly more often in hEDS and HSD compared to controls. Importantly, several symptoms/comorbidities occurred at a high level in both diagnoses including allergies, subluxations, sprains, headache, and migraine (16). However, we found that most symptoms/comorbidities differed between the two diagnoses. Patients diagnosed with HSD self-reported 42 conditions significantly more often compared to hEDS (42/115 or 37%), while only 9 conditions occurred more often in patients with hEDS (9/115 or 8%) (16). One important finding was that the conditions reported more often in hEDS were “structural” like prolapses whereas conditions reported more often in HSD were “functional” like irritable bowel syndrome. These findings indicate that key features of hEDS/HSD like mast cell activation/allergy, subluxations/sprains and headache/migraine are common to both diagnoses, but many other symptoms/comorbidities occur more often in patients with HSD. The structural issues in hEDS patients are not surprising, because the diagnostic criterion selects patients with these issues. What stands out is that this diagnostic “selection” results in patients with fewer systemic symptoms/comorbidities. Our findings of overlap in allergy/mast cell symptoms in hEDS and HSD are congruent with the recent report by Ritelli et al. who discovered plasma biomarkers in both hEDS and HSD, but not in controls, that are the product of extracellular matrix (ECM) degradation (collagen I and fibronectin fragments) (15). Mast cell enzymes are principally responsible for the breakdown of ECM proteins, and so high mast cell activity in hEDS and HSD patients could result in a similar plasma ECM degradation profile. Mast cell hyperactivation that degrades collagen and ECM provides a mechanism for hypermobility and similar symptoms/comorbidities between the diagnoses. Mechanisms to explain the differences between symptoms/comorbidities between the diagnoses are an avid area of research.

In contrast to our findings, several previous studies did not find a significant difference between symptoms and comorbidities between hEDS and HSD (Table 2) (9, 11–14). Ritelli et al. (20) conducted RNA sequencing of skin fibroblast biopsies from 20 patients with hEDS, 20 with HSD and 40 controls using the 2017 criteria, and found that the gene profiles overlapped between hEDS and HSD (Table 2) (20). In contrast, studies by Ritelli et al. have found differences in symptoms/comorbidities between hEDS and HSD with more symptoms in hEDS patients suggesting that they could be more severe than patients with HSD (8, 15). Several explanations for the differences between the studies include age or race differences and/or that Ritelli et al. reported data obtained from the medical record and our studies include self-reported data. Overall, most studies examined a relatively small number of symptoms/comorbidities in a small number of patients (Table 2). Perhaps a larger number of patients is needed before differences between the diagnoses appear. An oral abstract submitted to the *Ehlers-Danlos Society 2022 International Scientific Symposium on EDS and HSD* reported an initial analysis of the worldwide Ehlers-Danlos Society Registry that examined 8,867 hEDS and 990 HSD patients for 8 symptoms/comorbidities including fatigue, gastrointestinal symptoms, headache, anxiety, depression, dysautonomia, gynae, bladder and mast cell activation disease (9). They did not find any differences in these 8 categories of self-reported symptoms/comorbidities between patients with hEDS or HSD (9). However, the published abstract does not provide information on whether all patients were diagnosed using the 2017 criteria, information on age, race/ethnicity or statistical information. Thus, it is difficult to compare this large number of patients to our findings. We are currently examining whether specific comorbidities obtained from the medical record differ by diagnosis to determine whether self-reported differences match medical record data.

In this study we examined 2,695 patients using the 2017 diagnostic criterion with 60.6% (*n* = 1,632) diagnosed with HSD, 18.3% (*n* = 493) hEDS, 10.7% (*n* = 289) L-HSD or H-HSD, and 10.4% (*n* = 281) were controls without any of these diagnoses. This study has several advantages including a large number of patients diagnosed using the 2017 criterion, and a relatively large number of controls seen at the EDS Clinic that were not diagnosed with hEDS, HSD or L-HSD/H-HSD. Although the controls were older on average than the other groups, otherwise their demographic data was relatively similar, consisting primarily of non-Hispanic White women (Tables 3, 4). We found that hEDS, HSD and L-HSD/H-HSD patients consistently self-reported more symptoms/comorbidities than controls. Importantly, patients with L-HSD/H-HSD self-reported symptoms/comorbidities at levels that were very similar to HSD but differed from hEDS, as we previously reported (16). One possible explanation for the differences in our findings compared to the initial reports from the Ehlers-Danlos Society Registry is that our population is almost 90% White non-Hispanic and their study included many races/ethnicities. Importantly, our findings indicate that features specific to L-HSD/H-HSD such as hypermobility in other areas of the body, which are not included in the current diagnostic criterion, should be incorporated into future revised diagnostic criteria. Our findings also mirror patient experience, where many patients that do not receive a diagnosis according to the current 2017 diagnostic criterion indicate that they suffer from many of the same symptoms/comorbidities as hEDS and HSD patients, and they are upset that they have not received a diagnosis. Receiving a diagnosis not only confirms that patients’ suffering has a cause/explanation, but it is needed for patients to receive appropriate medical care. Gaining a better understanding of similarities and differences between patients with hEDS, HSD, L-HSD and H-HSD may improve patient care and provide mechanistic insight into the pathogenesis of disease. Research into these mechanisms may lead to improved tailored therapies that reduce the progression of disease.

There are several limitations to the current study. The site of this study is a tertiary care center and findings from patients in this study may not represent other regions of the US or world. Additionally, symptoms/comorbidities were self-reported and not validated through another method. Future studies are needed to examine whether the key findings of this study can be verified when medical records are examined. A strength of this study is that hEDS/HSD patients were diagnosed using the most recent 2017 criterion by physician experts. An additional strength is the large study population that contained an internal control group which was not diagnosed with hEDS, HSD, L-HSD/H-HSD but had the same diagnostic process.

In conclusion, the contribution of this study to the field is that most centers seeing EDS patients do not include L-HSD or H-HSD in a diagnosis of HSD so that these patients may not receive the same level of care as patients diagnosed with hEDS and HSD. Additionally, these patients may not be included in research studies of HSD and we found that these patients self-reported similar, and in some cases higher, levels of symptoms/comorbidities as HSD patients. Thus, our data suggests that the new diagnostic criteria should incorporate these patients into the criteria for HSD so that the patients receive care and research.

## Data Availability

The original contributions presented in the study are included in the article/supplementary material, further inquiries can be directed to the corresponding authors.
